# Environmental DNA detection of an invasive ant species (*Linepithema humile*) from soil samples

**DOI:** 10.1038/s41598-021-89993-9

**Published:** 2021-05-26

**Authors:** Tetsu Yasashimoto, Masayuki K. Sakata, Tomoya Sakita, Satoko Nakajima, Mamiko Ozaki, Toshifumi Minamoto

**Affiliations:** 1 Graduate School of Human Development and Environment, Kobe University, 3-11, Tsurukabuto, Nada-ku, Kobe, Hyogo 657-8501 Japan; 2 Research Fellow of Japan Society for the Promotion of Science, Tokyo, Japan; 3 Graduate School of Science, Kobe University, Kobe, Japan; 4 Kyoto Prefectural Institute of Public Health and Environment, Kyoto, Japan; 5 Graduate School of Engineering, Kobe University, Kobe, Japan; 6 KYOUSEI Science Center for Life and Nature, Nara Women’s University, Nara, Japan; 7 Research Institute of Sustainable Humanosphere, Kyoto University, Kyoto, Japan; 8 RIKEN Center for Biosystems Dynamics Research, Kobe, Japan

**Keywords:** Molecular ecology, Entomology, Biodiversity

## Abstract

Alien ant species (Formicidae, Hymenoptera) cause serious damage worldwide. Early detection of invasion and rapid management are significant for controlling these species. However, these attempts are sometimes hindered by the need for direct detection techniques, such as capture, visual observation, or morphological identification. In this study, we demonstrated that environmental DNA (eDNA) analysis can be used as a monitoring tool for alien ants using *Linepithema humile* (Argentine ant), one of the most invasive ants, as a model species. We designed a new real-time PCR assay specific to *L. humile* and successfully detected eDNA from the surface soil. The reliability of eDNA analysis was substantiated by comparing eDNA detection results with traditional survey results. Additionally, we examined the relationship between eDNA concentration and distance from nests and trails. Our results support the effectiveness of eDNA for alien ant monitoring and suggest that this new method could improve our ability to detect invasive ant species.

## Introduction

Alien ant species (Formicidae, Hymenoptera) have significant ecological and economic impacts^1–3^. Globally, they invade countries and regions that belong to both tropical and temperate zones^4–7^. They often dominate and eliminate native ants and other taxa (e.g., Hexapoda: Coleoptera, Lepidoptera, and Collembola, Mollusca: Panpulmonata, etc.)^8–10^ because of their common advantageous traits such as unicoloniality^11^ and polyphagia^12^. For example, a high population density caused by a unicolonial network called a supercolony allows them to overcome native organisms. Additionally, an increasing population of invasive ants in a supercolony can be dangerous for local people^13,14^ and can impact economic activities in agriculture, forestry, and fisheries^15,16^. For successful control of alien ants, similar to the control of other alien species, rapid detection of invasion and early-stage management is crucial^17–19^. Therefore, there is an urgent need to identify their invasion and distribution at the early stages. Traditionally, direct observations and/or bait trap surveys have been performed to monitor ant populations^20,21^. However, such methods have disadvantages such as the requirement for significant labor input, time, and expenditure. Moreover, these methods require technical expertise for the morphological classification of ant species, making it difficult for non-experts to obtain reliable results. For example, very closely related ant species sometimes only have few differences, such as the number of setae. This makes it challenging to discriminate between the alien species to be controlled and the native species to be conserved^22,23^, so that alien species may have become established within the native population by the time people perceive their invasion. For these reasons, it is desirable to develop rapid, easy, and reliable tools for monitoring alien ant species.

**Figure 1:**
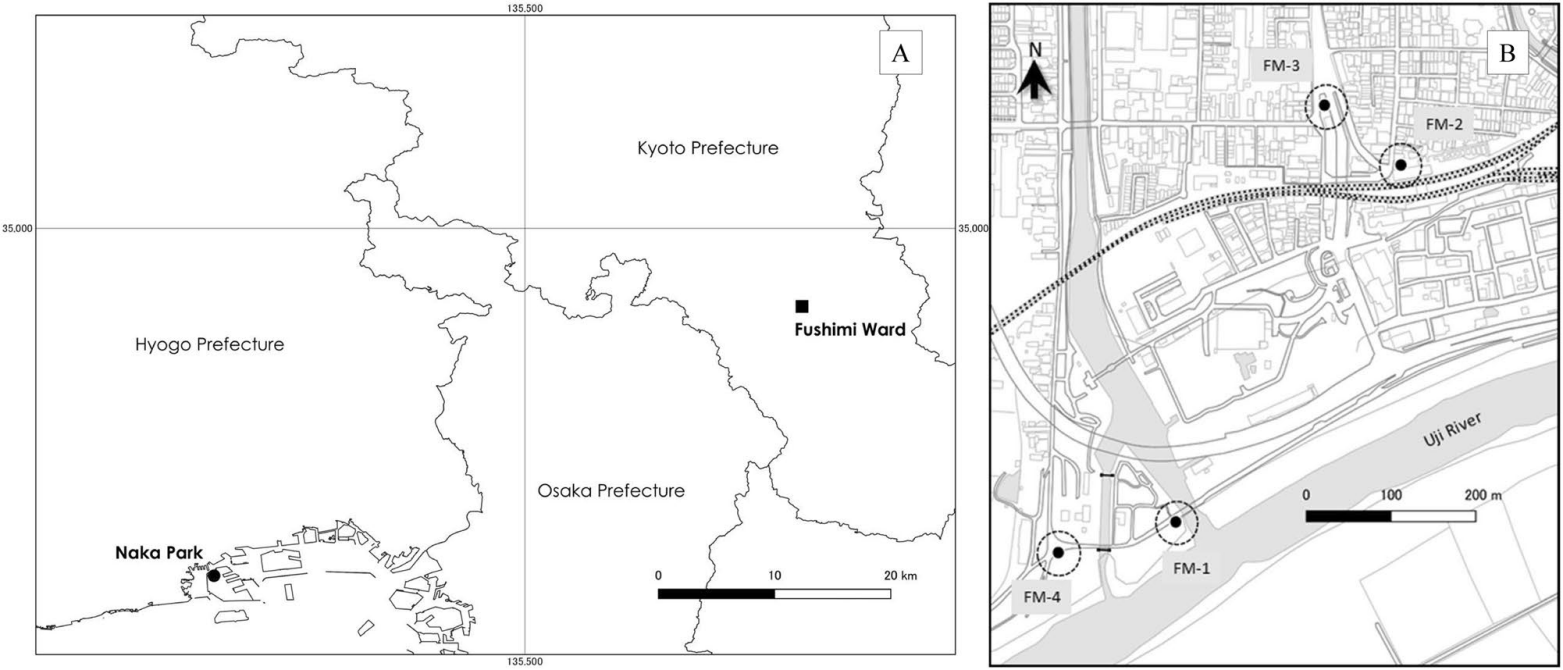
Locations of Naka Park and Fushimi Ward. (**A**) Each marker indicates the sampling sites (filled circle: Naka Park, Port Island, Kobe City and filled square: Fushimi Ward, Kyoto City). (**B**) Detailed map of the sampling area in Fushimi. The coordinates of the sampling sites are shown in Table S1. Figure S2 shows the sampling sites of Fushimi. This map was created using QGIS version 3.12.2 (http://www.qgis.org/) based on the Administrative Zones Data (http://nlftp.mlit.go.jp/ksj/gml/datalist/KsjTmplt-N03-v2_3.html) which were obtained from free download service of the National Land Numerical Information (http://nlftp.mlit.go.jp/ksj/index.html, edited by TY) provided by Japanese Ministry of Land, Infrastructure, Transportation and Tourism. There was no need of obtaining permissions for editing and publishing of map data.

*Linepithema humile* Mayr, 1868, also known as the Argentine ant was used as a model species in this study. This species is one of “100 of the world’s worst invasive alien species” listed by the International Union for Conservation of Nature and Natural Resources (IUCN). This South American ant species extends its distribution by forming huge single or multiple supercolonies in invaded areas. One of the largest supercolonies in Europe now covers 6000 km from Spain to Italy^24^. In Japan, it has caused serious damage, especially in western regions including Kyoto and Kobe^25–29^ (Fig. 1). These two areas have different management conditions for the control of *L. humile*, which have succeeded in Fushimi, Kyoto, but not in Port Island, Kobe. For Fushimi, the first invasion of *L. humile* was reported in 2008^30^. This densely settled residential area has suffered from the invasion of *L. humile*. Therefore, the Kyoto Prefectural Institute of Public Health and Environment has continued visual monitoring of *L. humile* using traditional bait traps since 2012. The invasive ants were controlled with poison bait and pesticide spray. Another area, Port Island, was invaded by this species in 1999^31^. There are four supercolonies of *L. humile* in Kobe, and two supercolonies, named “Kobe A” and “Kobe B”, in Port Island^27^. No management of the species was undertaken in this area, but the distribution of *L. humile* has been well studied^26,27^. Thus, with regard to special and historical interests, the sampling sites for the present study were selected from Fushimi and Port Island, respectively.

Environmental DNA (eDNA) analysis is a biological monitoring method that detects DNA in environmental samples, such as water, soil, or sediment^32–34^. The introduction of eDNA analyses can complement the limitations of traditional methods, which require real specimens and/or the direct observation of target species^35–37^. eDNA analysis has been used to detect various taxa and has been shown to be more sensitive than traditional methods^38–40^. For alien ants, as with other taxa, eDNA analysis may be either a superior or a complementary detection tool to traditional methods. However, most eDNA studies on macro-organisms target aquatic organisms, and few studies have focused on terrestrial organisms [for example^41^]. Basic information on eDNA concerning dispersal distance and persistence, relevant to the abundance or biomass, among other factors, has also been accumulated mainly in aquatic environments^42–45^, but not in terrestrial environments. Therefore, it is necessary to confirm whether eDNA analysis can detect alien ants from soil samples.

Our goal was to confirm the possibility of detecting eDNA from *L. humile* from soil samples and to investigate the applicability of eDNA analysis for alien ant monitoring. In this study, we (1) developed a real-time PCR assay specific to *L. humile*, (2) detected eDNA of *L. humile* from surface soil samples and compared it with traditional survey data, and (3) examined the relationship between eDNA concentrations and distances from their nests or trails to the soil sampling points.

## Results

### Designing a DNA detection assay specific to *L. humile*

We designed a pair of primers and a probe specific to *L. humile* (Table 1). Using an in silico test, we confirmed that the designed primers did not amplify any organisms other than *L. humile*. For the in vitro test, DNA was amplified only for *L. humile* and not for any non-target species (Table S2). There were no differences in the amplification of *L. humile* tissue DNA from the two supercolonies, Kobe A and Kobe B (p = 0.94, Welch's t test for Cq values). None of the negative controls showed any amplification signals. These results show that the primers and probe designed in this study were suitable for specific amplification of *L. humile* DNA in our study area, and that the amplification level was scarcely affected by genetic differences between supercolonies. This indicates that the newly designed detection assay is specific to *L. humile*. The limit of detection of the assay was 0.1 pg per reaction. We confirmed positive amplification of triplicates when 10, 1, and 0.1 pg/reaction of tissue-derived DNA was used as a template, but none of the triplicates were amplified when 0.01 pg a reaction was used.

**Table 1:** Sequences of designed primers and probe.

Lhu-CO1-F	5′-GATACACGAGCTTATTTCACATCTGC-3′	(Tm = 58.0 °C)
Lhu-CO1-R	5′-ATTGCTCATCAGAGAGTAGGGTTGT-3′	(Tm = 59.4 °C)
Lhu-CO1-P	5′-FAM-CTCTTCATGGAACTAAA-MGB-NFQ-3′	

“F” represents a forward primer, “R” represents a reverse primer, and “P” represents a TaqMan probe. The amplificon length was 128 bp.

### Detection of *L. humile* with traditional surveys in Fushimi

**Figure 2:**
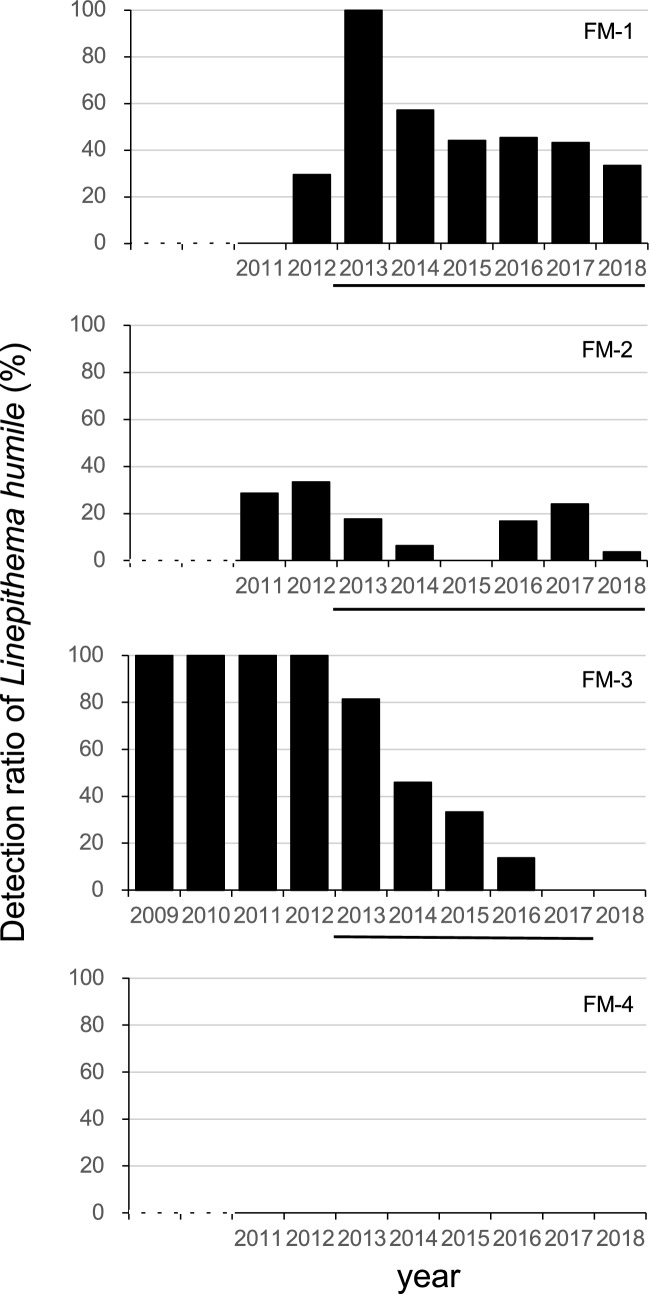
Annual change in the detection ratio of *L. humile* at Fushimi area. Bars indicate the period during which *L. humile* control was carried out.

At site FM-1, the detection ratio apparently decreased a year after the ant control started in December 2012 and subsequently decreased slightly (Fig. 2). The detection ratio at site FM-2 was not as high as that at sites FM-1 and FM-3. In 2015, *L. humile* was not detected but increased during 2016–2017 and then rapidly decreased in 2018. Thus, the variation in the detection ratio was because of the inconsistent thoroughness of the control and/or reinvasion of *L. humile*. At site FM-3, *L. humile* was observed quite frequently until ant control began in December 2012. The detection ratio then constantly decreased toward 2017 and reached a zero level—no *L. humile* has been found since.

A list of ant species captured and/or observed in 2018 within a radius of 50 m centered on the soil sampling points is shown in Table 2. Twenty ant species from four subfamilies were recorded in the four survey areas (see Fig. 1). Sites FM-1, FM-2, FM-3, and FM-4 had 7, 3, 15, and 10 species, respectively. The number of native ant species was low in the areas invaded by *L. humile*. This suggests that the invasion of *L. humile* had a significant negative impact on the native ant fauna of Fushimi.

**Table 2:** Ant species list observed with traditional surveys at Fushimi area in 2018.

	Subfamilies		FM-1	FM-2	FM-3	FM-4
	Species					
	**Ponerinae**					
1		*Pachycondyla chinensis* (*Emery*)		+	+	+
	**Myrmicinae**					
2		*Monomorium intrudens* Smith, F	+			
3		*Pheidole noda Smith, F*				+
4		*Tetramorium tsushimae* Emery	+		+	+
5		*Crematogaster* (*Crematogaster*) *matsumurai* Forel		+	+	+
6		*Crematogaster* (*Orthocrema*) *osakensis* Forel	+			+
7	*	Temnothorax congruus (Smith, F.)			+	
8	*	*Temnothorax spinosior* (Forel)			+	+
9		*Temnothorax mitsukoae* Terayama & Yamane			+	
10		*Solenopsis japonica* Wheeler, W.M	+		+	
11	*	*Pristomyrmex punctatus* (Smith, F.)			+	+
	**Dolichoderinae**					
12		*Dolichoderus sibiricus* Emery			+	
13		*Ochetellus glaber* (Mayr)			+	
**14**	*	***Linepithema humile* (Mayr)**	** + **	** + **		
	**Formicinae**					
15	*	*Formica japonica* Motschoulsky	+			+
16		*Lasius (Lasius) japonicus* Santschi			+	
17		*Nylanderia flavipes* (Smith, F.)	+		+	+
18		*Nylanderia amia* (Forel)			+	
19	*	*Prenolepis* (*Nylanderia*) *sakurae Ito*			+	+
20	*	*Camponotus vitiosus* Smith, F			+	
		Number of species	7	3	15	10
		Detection ratio of *L. humile*	33%	4%	0%	0%
		Number of research surveys	36	83	23	30

Species shown in bold letters represent the target species (*Linepithema humile*). “+” is marked in the box for species that were detected at least once. *Overlapping species that were also found on Port Island, Kobe. “Number of species” includes *L. humile.*

### eDNA detection from soil and comparison with data from traditional methods

DNA amplification was confirmed in samples from sites FM-1 and FM-2, where live *L. humile* were found at the time of soil sampling and 2 weeks before sampling, respectively (Table 3, Table S3). By direct sequencing, all of the amplified DNA was confirmed to be derived from *L. humile*. eDNA was not detected in samples from sites FM-3 and FM-4, where *L. humile* had not been detected at any time since 2017 and 2011, respectively (Table 3, Table S3). DNA was not amplified in any negative control.

**Table 3:** Comparison of the results of eDNA analysis using samples from Fushimi and traditional survey methods.

Sampling site	eDNA	Traditional	Visual
FM-1	+(3/3, Cq: 36.94 ± 0.05) +(3/3 Cq: 39.62 ± 0.22) +(2/3, Cq: 40.97)	+	+
FM-2	+(3/3, Cq: 39.67 ± 00.12) +(2/3, Cq: 39.14) +(2/3, Cq: 41.43)	+	–
FM-3	– – –	–*	–
FM-4	–	–	–

The column “eDNA” indicates whether eDNA was detected by real-time PCR. The numbers in parentheses are the number of positive replicates and the average and standard error of Cq values (if positive in 2/3, only the average is shown). The column “Traditional” shows data from the regular visual survey of *L. humile* conducted by the Kyoto Prefectural Institute of Public Health and Environment, and * indicates the site where individuals were observed until 2 years ago but where they have not been observed in the last 2 years. The column “Visual” indicates whether *L. humile* individuals were visually observed on the day of soil sampling. The raw data are presented in Table S4.

### Relationship between eDNA and distance from the nest or trail

In the soil samples collected in October 2017, we detected *L. humile* DNA in all samples, even though neither nests nor foraging workers of *L. humile* were found around the PI-D sampling point (Table 4). No eDNA amplification was observed in any of the negative controls.

**Table 4:** Real-time PCR results for Port Island October 2017.

Series	Sample name	Number of positive replicates	Cq ± SE
A (nest)	A0	3/3	28.83 ± 0.07
	A10	3/3	32.14 ± 0.04
	A50	3/3	31.85 ± 0.06
	A100	3/3	34.30 ± 0.06
B (trail)	B0	3/3	38.61 ± 0.27
	B10	3/3	40.38 ± 0.51
	B50	3/3	39.86 ± 0.43
	B100	3/3	35.90 ± 0.11
C	C	3/3	28.37 ± 0.09
D	D	3/3	29.33 ± 0.01

The column “Cq” shows the average Cq values among the three replicates in real-time PCR. The numbers in each sample name in series A and B represent the distance (cm) from the nest or trail. Sample C was from a pile beside a *L. humile* nest, and sample D was soil at the site where *L. humile* was not found. The raw data are listed in Table S5.

The eDNA concentration, as determined by Cq values, showed no statistical relationship with distance from the nest in the two series (p = 0.156, GLMM, Fig. S1A; p = 0.255, GLMM, Fig. S1B).

**Figure 3:**
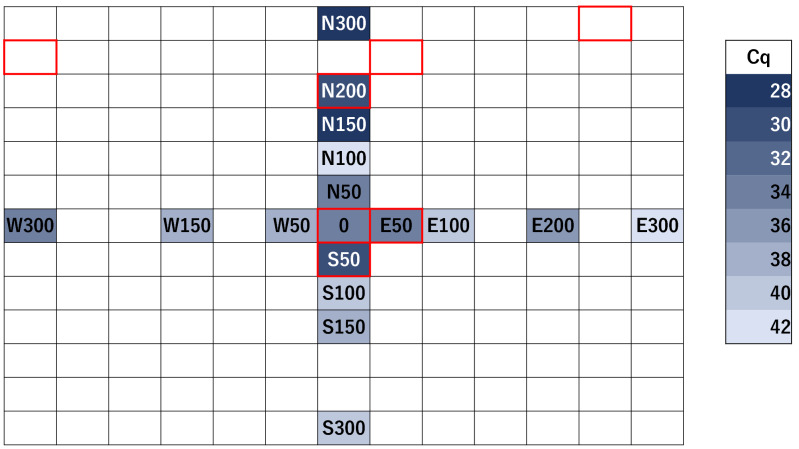
Comparison of Cq values obtained by real-time PCR of Port Island samples in November 2018. The cells were colored based on the Cq value of each sample. The darker blue indicates lower Cq value, indicating more DNA. Red frames indicate areas where *L. humile* individuals or nests were found. Samples of S200, E150, W100, and W200 were excluded because DNA could not be extracted successfully (see text for details).

eDNA was detected in 17 of the 21 samples collected in November 2018 from the expanded sampling area up to 300 cm from the nest (Table 5, Fig. 3). Cq values were small, where *L. humile* forager workers were frequently seen at or beside the sample point (PI-N150, PI-N200, PI-N300, PI-S50, and PI-E50). The pattern of changes in Cq values was different in each direction: increased with distance from the nest in two directions (S and E) but had no pattern in the other two directions (N and W).

**Table 5:** Real-time PCR results for Port Island November 2018.

Series	Sample name	Number of positive replicates	Cq $\pm \mathrm{SE}$
N	0	3/3	34.61 ± 0.05
	N50	3/3	35.96 ± 0.05
	N100	3/3	42.26 ± 1.09
	N150	3/3	29.84 ± 0.02
	N200	3/3	30.66 ± 0.02
	N300	3/3	28.36 ± 0.02
S	0	3/3	34.61 ± 0.05
	S50	3/3	30.44 ± 0.16
	S100	3/3	40.24 ± 0.54
	S150	3/3	39.74 ± 0.45
	–	–	–
	S300	3/3	41.12 ± 0.10
E	0	3/3	34.61 ± 0.05
	E50	3/3	34.80 ± 0.17
	E100	3/3	41.39 ± 0.81
	–	–	–
	E200	3/3	36.45 ± 0.07
	E300	2/3	43.29
W	0	3/3	34.61 ± 0.05
	W50	3/3	39.56 ± 0.11
	–	–	–
	W150	3/3	38.31 ± 014
	–	–	–
	W300	3/3	34.41 ± 0.07

Sample 0 in each series was collected beside the reference nest and was common to all series. The letters represent their series for all other sample names, and the numbers represent the distance from the nest (cm). The column “Cq” represents the average of the Cq values among three PCR replicates (the average of the two positive replicates for E300). Samples from S200, E150, W100, and W200 were excluded because DNA could not be extracted successfully (see text for details). The raw data are listed in Table S6.

One of the four extraction negative controls prepared with the samples from Port Island in November 2018 was positive (1/3 replicates). However, a positive result was obtained in one of the 12 replicates of extraction negative controls, and it was judged to have no effect on the experimental results.

## Discussion

### eDNA detection assay for alien ant species

The target region for the primers and probe that we designed was mitochondrial cytochrome c oxidase subunit 1 (CO1), which is generally used for the barcode of life. We were able to obtain a consensus sequence of this region for *L. humile* by referring to a large number of sequences in a substantial database (Table S7). However, for several co-existing non-target species (i.e., *Crematogaster matsumurai*, *Monomorium chinense*, *Paratrechina amia*, *Paratrechina sakurae*, and *Temnothorax congruus*), reference data were not found. Considering the results of the in silico and in vitro tests, our new detection assay is suitable for the survey of *L. humile* in Japan. However, the lack of DNA sequence data for many other ant species in the world may prevent future eDNA studies targeting other alien ants or applying them to other areas. The reference DNA database of diverse ant species should be promptly improved to establish a method for detecting alien ants using eDNA analysis.

To enhance the detection power of *L. humile* eDNA, we adopted the DNA extraction method described by Sakata et al*.*^46^ rather than the original protocol for the commercial kit. We increased the soil sample volume up to 3 g, although the kit's standard sample volume is 0.25 g, and obtained positive signals from most of our samples. However, we found that the volume of pellets produced by ethanol precipitation was larger in our terrestrial soil samples than in the underwater sediment samples collected by Sakata et al*.*^46^. As a result, the tube was sometimes full when pellets were placed into the PowerSoil DNA Isolation Kit Power Bead Tube. Thus, reagents may not have been sufficiently mixed with the sample and DNA could not be successfully extracted, which was the case with some samples (PI-S200, PI-W100, PI-W200, and PI-E150). This was probably because of the difference in the water content between the samples. The amount of soil (or sediment) contained in the same 3 g samples may have differed between the sample types. For wider application, additional modification in the experimental procedure might be needed, case-by-case, according to specific requirements in each sample type.

### Reliability and applicability of eDNA analysis for alien ants

For the Fushimi samples, the results of the traditional observation and eDNA analyses were in complete agreement (Table 3). Positive eDNA signals were detected for site FM-1 but not for FM-4, in accordance with the *L. humile* workers that were observed in the FM-1-sampling site but never in the FM-4. There was inconsistency in the results from FM-2 between eDNA detection and the visual survey (Table 3, Table S3). However, considering that a large number of individuals had been found a week before and the visibility of FM-2 was poor owing to the lack of any management such as mowing (Fig. S2B), it should be regarded as a false negative in the visual survey. Similarly, the detection of eDNA from sample PI-D, where no individuals were found at the time of sampling, could also be the effect of overlooked individuals. Additionally, *L. humile* always moves along trails for foraging or changing their nest site. Their trails toward better food or residences frequently appear and disappear because food sources and nests are non-permanent. Thus, unless the *L. humile* eDNA quickly degrades on the ground surface, eDNA analysis can detect the target species even in the absence of workers or their trails around the sampling site, as was the case in sites FM-2 and PI-D. These results suggest that eDNA analysis is effective in terrestrial environments where visual observation is difficult, as well as in aquatic environments.

No amplifications were found in any of the nine replicates of FM-3 (three sampling replicates and three PCR replicates per sample), where *L. humile* has not been observed for 2 years (Fig. 2, Table 3). In this area, reinvasion of *L. humile* has been successfully prevented for 2 years after eradication. This suggests that DNA in the soil surface layer was degraded within a maximum period of 2 years, eliminating the possibility of false positives owing to residual DNA. For comparison, in lake sediments, DNA is protected from degradation by adsorption in sediment particles^36,47^. It is expected that DNA on the soil surface could persist for a long time, although it may not be as long as that observed for underwater sediment^48^. A recent study by Foucher et al*.*^49^ showed that eDNA cultivated at 20–30 cm underground might remain for 6 or 7 years. Our results showed that the persistence time scale of eDNA at the soil surface was relatively shorter, possibly because the absorption of soil particles, weathering, and UV effects at the soil surface may have promoted DNA degradation. The persistence of eDNA in soil likely differs according to soil properties, particularly soil moisture, temperature, and organic carbon content^50^. In other words, our study provided important insights; however, further studies are needed.

Concerning the concentration of eDNA, the results of FM-1 (average Cq = 38.95 ± 0.60) and PI-A0 (average Cq = 32.14 ± 0.04), both of which were collected beside the nest of *L. humile*, showed large differences in Cq values (Tables 3, 4). This may depend on soil properties that act as detection inhibitors and degradation factors, which can result in differences in DNA recovery rates by affecting DNA binding and release^51–53^. This suggests that, combined with the differences in persistence mentioned above for soil eDNA, comparison of Cq values or eDNA concentration is only possible among samples from the same location and may be difficult among samples from different sites. Otherwise, the difference in Cq values could reflect the colony size. Quantitative estimation of colony size using eDNA analysis remains untested.

### Relationship between DNA concentration and distance

Using the soil samples from Port Island in October 2017 and November 2018, we examined the relationship between eDNA concentration in soil samples and the distance from ant nests or trails to the sampling points, but there were no clear or consistent patterns. Although intuitively, we would expect the number of ants and hence eDNA to have a negative relationship with distance from a nest, this simple relationship would likely be confounded when there are multiple nest entrances within a sample area, and when there are multiple foraging trails that change regularly.

Notably, sample PI-C, which was a refuse pile of dirt from nest excavations beside the nest entrance, showed the lowest Cq value (i.e., detected the highest eDNA concentration) in our dataset (Table 4). This indicates that different ant activities within the substrate will result in different eDNA being present. More details regarding the relationship between ant behavior and remaining eDNA should be clarified in future studies.

### Availability of eDNA analysis for controlling or monitoring invasive ants

In this study, we developed a specific real-time PCR assay for *L. humile* DNA and successfully detected the eDNA of *L. humile* from surface soil samples. We also compared the results of eDNA detection with those of a traditional survey and found that the eDNA analysis provided reliable data. In our preliminary comparison between our real-time PCR method and the LAMP method, the real-time PCR method might have approximately 1000 times higher detection sensitivity than the LAMP method (see Table S8). Furthermore, this is the first attempt to consider the relationship between eDNA concentration and distance from ant nests and trails. The results of this study suggest that eDNA analysis is useful for supporting or complementing traditional methods, especially in cases where visual observation is difficult. These findings are applicable to other alien ant species. Additionally, although ant species are generally difficult to classify morphologically because of their large number of species, small body size, and close resemblance of morphological characteristics^22,23,54^, eDNA analysis can provide accurate identification based on genetic information. These results support the hypothesis that eDNA analysis is a suitable tool for the detection of alien ants.

We demonstrated that eDNA analysis can be an effective tool for monitoring alien ant species. However, there are still some points that need to be clarified before the method of detecting eDNA of ant from soil samples can be applied to actual invasive ant control. For example, it is not known how individuals release their DNA into the environment, what their eDNA dynamics in the soils are and, therefore, how far from the nest eDNA can be detected. In addition, similar to what is known for eDNA in water^55^, the seasonality of eDNA concentration is expected to affect the detection probability. It should also be noted that the positive eDNA detection does not directly indicate the presence of live individuals, and we should consider the DNA persistence period in soil. Moreover, we need to know how many samples are needed to survey a given area. Finally, differences in DNA extraction efficiency owing to differences in the nature of each soil sample should also be considered. Some of these points can be clarified with laboratory assays, and some require field surveys. Such further efforts will help to generalize the sampling strategy. If these points are clarified and an optimal sampling scheme is established, it may be possible to use eDNA analysis to estimate a range of activities of alien ants in greater spatial detail. In the future, the advancement of basic and applied studies of terrestrial eDNA may lead to early detection and control of alien ant species, combined with traditional survey methods. The introduction of eDNA analysis to alien ant monitoring is helpful for accurate, rapid, and efficient surveys.

## Methods

### Designing primers and a probe and tests for specificity

The target region for primers and a probe was the mitochondrial CO1, which is commonly used for DNA barcoding in animals. Referring to Sunamura et al*.*^27^, CO1 sequences of 10 species, *L. humile*, and nine other species found on Port Island, were downloaded from the National Center for Biotechnology Information (NCBI) (Table S7). We then aligned them and designed a pair of primers for *L. humile*, Lhu-CO1-F, and Lhu-CO1-R, to contain bases specific to the target species within five bases at the 3' end (Table 1). An in silico test using Primer-BLAST (https://www.ncbi.nlm.nih.gov/tools/primer-blast/index.cgi) with default settings was performed to confirm specificity. We also designed a probe for *L. humile*, Lhu-CO1-P, using Primer Express Software 3.0 (Applied Biosystems, Foster City, CA, USA) with default settings (Table 1).

On August 3, 2018, we sampled several individuals, including *L. humile* and nine non-target species found on Port Island (*Camponotus vitiosus*, *Formica japonica*, *Monomorium chinense*, *Paratrechina flavipes*, *Paratrechina sakurae*, *Pristomyrmex punctatus*, *Temnothorax congruus*, *Temnothorax spinosior*, and *Tetramorium tsushimae* (Fig. 1). Several workers of each species were captured with tweezers and immediately stored in vials containing 70% ethanol. We brought them back to the laboratory where they were morphologically identified. For *L. humile*, we collected worker ant samples that appeared to be from two different supercolonies (Kobe A and Kobe B), referring to the distribution map on Port Island^27,56^. Then, aggression behavior assays of workers were used to confirm which supercolonies the tested worker belong to—Kobe A or Kobe B^56^, using individuals kept in our laboratory from each of the supercolonies. In short, we placed each individual sample on the trail of *L. humile* from both supercolonies. If individuals exhibited aggressive behavior, they were regarded as belonging to a different supercolony.

DNA was extracted from the tissues of individual samples using the DNeasy Blood & Tissue Kit (QIAGEN, Hilden, Germany). First, the whole body of each *L. humile*, Kobe A and Kobe B, and non-target species were separately mashed with BioMasher II (EOG-sterilized, Nippi, Tokyo, Japan). Subsequent extraction methods followed QIAGEN's Quick-Start protocol to obtain 200 µl of tissue DNA samples for each specimen.

Tissue DNA from each specimen was used as a template for real-time PCR to confirm the specificity of the designed primers and a probe. Real-time PCR was performed using a CFX96 Touch Real-Time PCR Detection System (Bio-Rad, Hercules, CA, USA). The PCR mixture was prepared at 20 µl per well, including 1 × TaqMan Environmental Master Mix 2.0 (Life Technologies, Foster City, CA), 900 nM of each primer, Lhu-CO1-F and Lhu-CO1-R, 125 nM TaqMan MGB probe, Lhu-CO1-P, 0.1 µl AmpErace Uracil *N*-glycosylase (Thermo Fisher Scientific, Waltham, MA, USA), 2 µl DNA sample (5 pg/µl), and ultrapure water. PCR-negative controls were prepared using ultrapure water instead of DNA sample solutions. PCR conditions were as follows: an initial step at 50 °C for 2 min and 95 °C for 10 min, followed by 55 cycles of 95 °C for 15 s and 60 °C for 1 min. PCR was performed in triplicate for all samples and negative controls. To check whether the difference in supercolonies affected the amplification efficiency, the Cq values (N = 3 for each individual) of real-time PCR using DNA from an individual of each supercolony were compared using Welch’s t test.

The limit of detection for the assay was tested using a dilution series of tissue-derived DNA. Real-time PCRs were performed using 10, 1, 0.1, and 0.01 pg per reaction of template DNA in triplicate as described above.

### Traditional *L. humile* distribution surveys and control

In Fushimi, traditional *L. humile* surveys were conducted at four sites: FM-1, 2, 3, and 4 (Fig. 1, Fig. S2, Table S3). The surveys were started in 2009 (FM-3) or 2011 (the other three sites) and continued until 2018. Our study site is a residential area and a large proportion of it is paved; therefore, we used bait traps in this study instead of other traditional methods such as pitfall traps. Traditional trap surveys^57^ were performed in all sampling areas, and observations and hand sampling^58^ were conducted at FM-3. For the trap survey, we used two types of traps. One was a PP trap (#54035250, Kankyokiki Co., Ltd., Osaka, Japan), made of polypropylene with an 8 cm square area sticky surface, and the other was a sugar trap made of a cotton pad (6 cm × 5 cm) soaked in 30% w/v sugar solution. We set up PP traps four times a year (twice for FM-3) and sugar traps monthly. PP traps and sugar traps were collected 3 days and 30 min after setting, respectively. The captured individuals were morphologically identified. At FM-3, ants were observed and captured for 15–20 min. The number of surveys conducted by each method at each site is listed in Table S9. The detection rate was defined as the percentage of times *L. humile* was captured out of the total number of times surveyed per year for each survey site.

### Detection of eDNA from field samples

On October 12, 2018, 10 surface soil samples were collected at four sites in Fushimi (n = 3 in FM-1, FM-2, and FM-3, and n = 1 in FM-4, Fig. 1, Fig. S2, Table S3). Approximately 25 g of each soil sample was collected and placed into 50 ml tubes with rubber-gloved hands or a disposable shovel, and then stored at − 25 °C until DNA extraction. Care was taken not to put the individual ants, pebbles, and leaves into the tube. We conducted visual surveys for 30 min, at an approximately 10 m or more radius around each sampling site with two experts looking for *L. humile*.

Similar to the DNA extraction method from aquatic sediments described in a previous study^46^, DNA extraction was performed using a combination of alkaline DNA extraction^59^, ethanol precipitation, and PowerSoil DNA Isolation Kit (MO Bio Laboratories, Carlsbad, CA, USA). This process was performed in a dedicated room for eDNA experiments, which were separated from the room where tissue DNA extraction or PCR was performed. We prevented DNA cross-contamination by wearing disposable rubber gloves during all experiments and by using washed consumables with chlorine bleach (0.1% effective chlorine concentration)^60^. First, 3 g of soil sample was placed in a 15 ml tube and vortexed with 6 ml NaOH (0.33 M) and 3 ml Tris‐EDTA buffer (pH 6.7). After incubating at 94 °C for 50 min (lightly shaking about every 15 min), it was cooled to room temperature and centrifuged at 5000×*g* for 30 s. Then, 7.5 ml of supernatant was transferred to a 50 ml tube and neutralized with an equal volume of Tris–HCl (1 M, pH 6.7). Next, 1.5 ml sodium acetate solution (3 M, pH 5.2) and 30 ml absolute ethanol were added, vortexed, and stored at − 25 °C for more than 1 h (ethanol precipitation). The sample was then centrifuged at 5350×*g* for 20 min, and the supernatant was discarded. The precipitate content in the tube was transferred to a Power Bead Tube (PowerSoil DNA Isolation Kit). The residue was dissolved in 100 µl of ultrapure water and transferred to the same power bead tube. The following steps were performed according to the Experienced User Protocol 3 to 22 of the PowerSoil DNA Isolation Kit. Negative controls were prepared using ultrapure water of the same mass instead of soil samples. The resulting soil-derived eDNA samples were stored at − 25 °C.

Real-time PCR was performed under the same conditions and reagent compositions as described above. In addition to soil DNA samples (n = 10) and extraction negative controls, PCR-positive controls and PCR-negative controls were amplified in triplicate. For the PCR positive control, the tissue DNA of *L. humile* Kobe A (5 pg/µl) prepared as mentioned above was used as a template. The amplified products were directly sequenced using a commercial sequencing service (Fasmac Co., Ltd., Atsugi, Japan).

### Investigation of the relationship between eDNA concentrations and distance from ant nests or trails

On October 13, 2017, in Naka Park, Port Island (Fig. 1), an additional 10 surface soils were sampled at four distances from the nests or trails of *L. humile*. We hypothesized that eDNA in soil does not spread as much as in water; therefore, we set the maximum distance to 100 cm. (1) A0, A10, A50, and A100 were sampled at distances of 0, 10, 50, and 100 cm from *L. humile* nests, respectively; (2) B0, B10, B50, and B100 were sampled at the same distances from their trail, (3) C was a sample from a part of the pile beside the nest, and (4) D was a soil sample from a location where no *L. humile* was seen at the time of sampling. eDNA was extracted from the soil samples and amplified in the same way as described above. The same series of eDNA samples were run on a single PCR plate to compare the Cq values.

A generalized linear mixed model (GLMM, Gaussian distribution) was used with the lmer function in the glmer test package of R version 3.5.1^61^ for correlations between the amount of eDNA detected and the distance from nest or trail. Cq values resulting from real-time PCR were used as reciprocal indicators of the amount of eDNA (i.e., the higher the eDNA concentration in the sample, the smaller the Cq value). GLMM was calculated by setting the distance from the nest or trail as an explanatory variable, the Cq value as a response variable, and PCR replicates as a random term.

Based on the results of the above experiments and analysis, soil sampling with a wider sampling scale (at most 300 cm from a nest) was carried out. We collected soil samples from Naka Park, Port Island, from the ground surface around a nest on November 19, 2018. One sample was right beside the nest, and an additional 20 samples were also collected at five distances (50, 100, 150, 200, and 300 cm) in four directions (N, E, S, W) from a reference nest, giving a total of 21 samples. The sampling method was the same as described above. At the same time, visual observation was also conducted within a 5 m radius around the nest, and the locations of other *L. humile* nests and individuals were recorded. DNA extraction and amplification were performed, but in some samples, sufficient precipitation did not occur after centrifugation in step 7 of the PowerSoil DNA Isolation Kit Experienced User Protocol. Real-time PCR was performed as described above, and the same series of eDNA samples were run on a single PCR plate to compare the Cq values. Samples were tested for PCR inhibition because DNA was not detected in these samples. All samples were amplified in the same way as above, except that 2 µl of tissue DNA of target species per well was added instead of reduced ultrapure water. Therefore, we confirmed that there were no PCR inhibitory effects in any of the samples. For this reason, S200, W100, W200, and E150 were considered as failed DNA extractions and were excluded from the results.

## Supplementary Information

Supplementary Information 1.
