# Supplementary material for: Environmental DNA detection of an invasive ant species (Linepithema humile) from soil samples
Source: Sci Rep. 2021 May 26;11:10712. doi: 10.1038/s41598-021-89993-9 (PMC8154996; doi:10.1038/s41598-021-89993-9)
Supplement: Supplementary file 1 — Supplementary Information 1. [file 41598_2021_89993_MOESM1_ESM.docx]

**Supplementary information for:**

**Environmental DNA detection of an invasive ant species (*Linepithema humile*) from soil samples.**

Tetsu Yasashimoto ^a^, Masayuki K. Sakata ^a, b^, Tomoya Sakita ^c^, Satoko Nakajima ^d^, Mamiko Ozaki ^c, e, f, g, h^, Toshifumi Minamoto ^a^,

^a^ Graduate School of Human Development and Environment, Kobe University, Kobe, Japan

^b^ Research Fellow of Japan Society for the Promotion of Science, Tokyo, Japan

^c^ Graduate School of Science, Kobe University, Kobe, Japan

^d^ Kyoto Prefectural Institute of Public Health and Environment

^e^ Graduate School of Engineering, Kobe University, Kobe, Japan

^f^ *KYOUSEI* Science Center for life and Nature, Nara Women’s University, Japan

^g^ Research Institute of Sustainable Humanosphere, Kyoto University, Kyoto, Japan

^h^ RIKEN Center for Biosystems Dynamics Research, Kobe, Japan


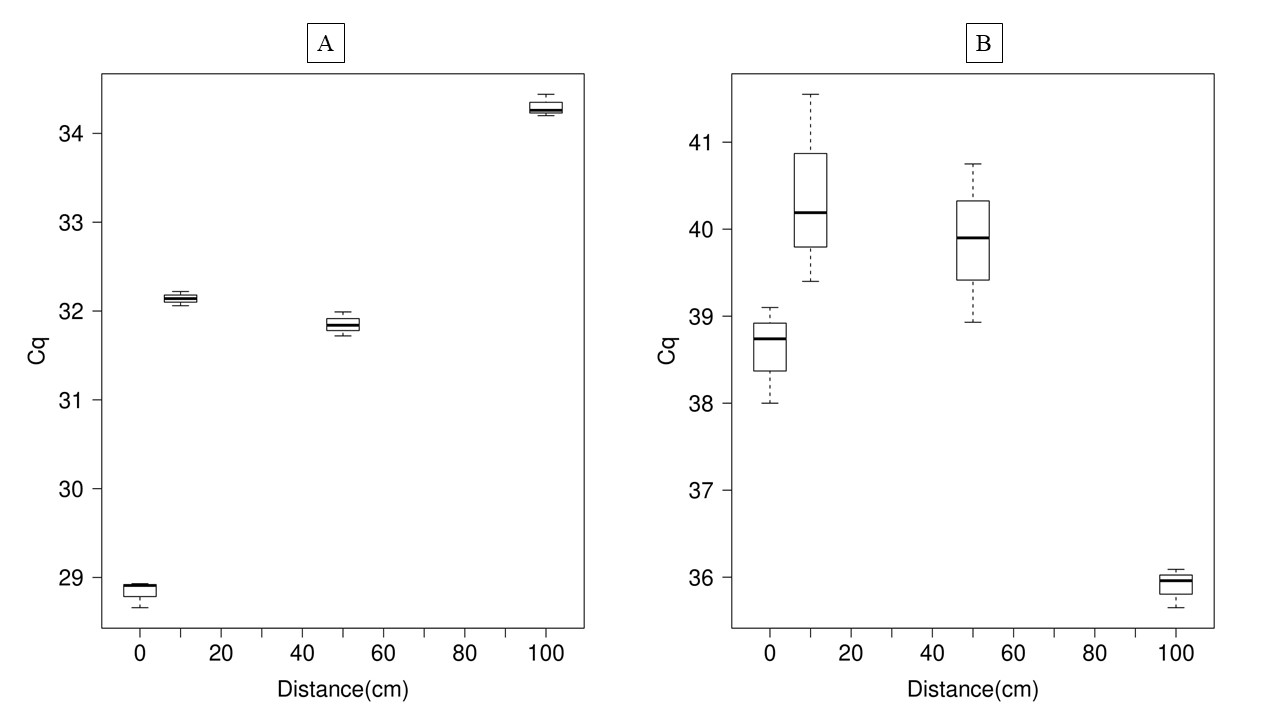
 Figure S1: The relationship between distance and Cq values of Port Island samples in October 2017.

The horizontal axis shows the distance from the nest (A) or trail (B). The vertical axis shows the Cq values of the samples

(A) FM-1


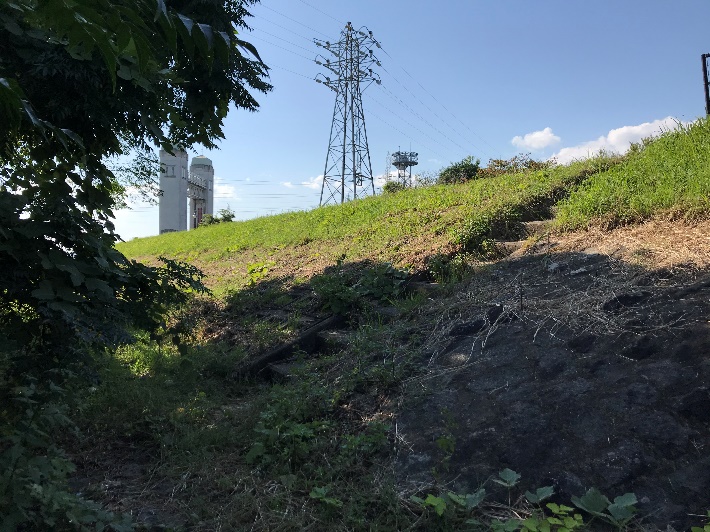


(C) FM-3


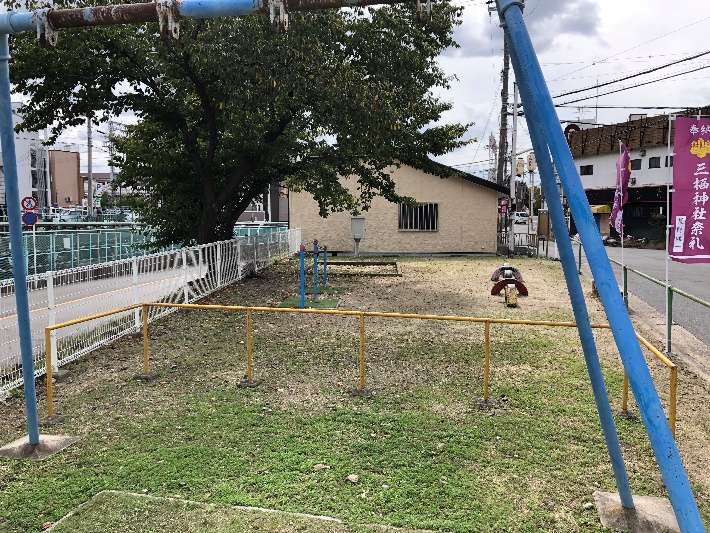


Figure S2. View of the Fushimi sampling site

(B) FM-2


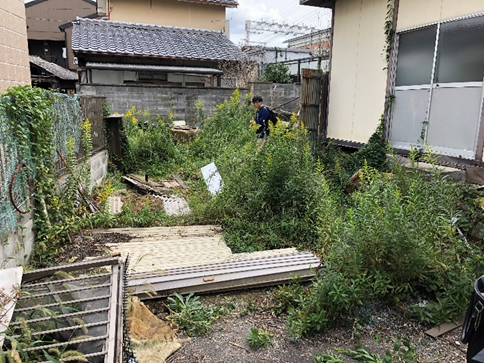


(D) FM-4


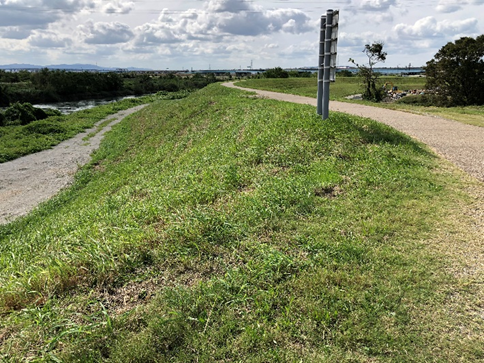


Table S1. Coordinate of sampling site, Fushimi, Kyoto and Naka Park, Port Island, Kobe

| Sampling site | | north latitude | east longitude |
| --- | --- | --- | --- |
| Fushimi, Kyoto | FM-1 | 34.92333 | 135.75700 |
|  | FM-2 | 34.92697 | 135.75990 |
|  | FM-3 | 34.92758 | 135.75891 |
|  | FM-4 | 34.92302 | 135.75549 |
| Port Island, Kobe | Naka Park | 34.673951 | 135.208214 |

Table S2. Results of real-time PCR to check specificity

| Species | Temp. DNA conc. | Number of positive replicates | Cq ±SE |
| --- | --- | --- | --- |
| *Linepithema humile* (Kobe A) | 10pg/well | 3/3 | 29.51 ±0.03 |
| *Linepithema humile* (Kobe B) |  | 3/3 | 28.22 ±0.004 |
| *Camponotus vitiosus* |  | 0/3 | – |
| *Formica japonica* |  | 0/3 | – |
| *Monomorium chinense* |  | 0/3 | – |
| *Paratrechina flavipes* |  | 0/3 | – |
| *Paratrechina sakurae* |  | 0/3 | – |
| *Pristomyrmex punctatus* |  | 0/3 | – |
| *Temnothorax congruus* |  | 0/3 | – |
| *Temnothorax spinosior* |  | 0/3 | – |
| *Tetramorium tsushimae* |  | 0/3 | – |

The column for DNA concentration shows the amount of DNA added to each well in real-time PCR, and the column for Cq value shows the average of the Cq values among the replicates that were positive. The mark “–” in the column “Cq” represent that Cq values couldn’t be calculated because of no positive signals of DNA.

Table S3. Details of sampling locations and sample size in Fushimi, Kyoto

| Sampling site | Site detail | n |
| --- | --- | --- |
| FM-1 (Uji-river riverbed) | The first confirmed records of invasion by Argentine ant was in October 2012. At present, this site is located at the boundary of invasion and non-invasion at Fushimi. | 3 |
| FM-2 (An abandoned house site) | A large number of Argentine ants were found a week before the soil sampling date. | 3 |
| FM-3 (Children’s playground) | The area where the eradication has been completed. Argentine ants were seen in large numbers until two years ago, but they have never been found since then. | 3 |
| FM-4 (Uji-river riverbed) | Argentine ants have never been found. | 1 |

Column “n” represents the number of soil samples collected from each site for eDNA analysis.

Table S4. Amplification detail of Fushimi Oct. 2018

| Sampling site | Sampling replicates | PCR replicates | Cq |
| --- | --- | --- | --- |
| FM-1 | 1 | 1 | 41.69 |
|  |  | 2 | 40.25 |
|  |  | 3 |  |
|  | 2 | 1 | 36.90 |
|  |  | 2 | 37.05 |
|  |  | 3 | 36.86 |
|  | 3 | 1 | 39.52 |
|  |  | 2 | 39.21 |
|  |  | 3 | 40.13 |
| FM-2 | 1 | 1 | 39.36 |
|  |  | 2 | 39.84 |
|  |  | 3 | 39.80 |
|  | 2 | 1 | 42.11 |
|  |  | 2 | 40.75 |
|  |  | 3 |  |
|  | 3 | 1 | 38.90 |
|  |  | 2 | 39.39 |
|  |  | 3 |  |
| FM-3 | 1 | 1 |  |
|  |  | 2 |  |
|  |  | 3 |  |
|  | 2 | 1 |  |
|  |  | 2 |  |
|  |  | 3 |  |
|  | 3 | 1 |  |
|  |  | 2 |  |
|  |  | 3 |  |
| FM-4 |  | 1 |  |
|  |  | 2 |  |
|  |  | 3 |  |
| PCR-PC |  | 1 | 29.22 |
|  |  | 2 | 29.15 |
|  |  | 3 | 28.98 |
| Ex-NC |  | 1 |  |
|  |  | 2 |  |
|  |  | 3 |  |
| PCR-NC |  | 1 |  |
|  |  | 2 |  |
|  |  | 3 |  |

Table S5. Amplification detail of Port Island Oct. 2017

| Sample name | PCR replicates | Cq |
| --- | --- | --- |
| A0 | 1 | 39.10 |
|  | 2 | 38.00 |
|  | 3 | 38.74 |
| A10 | 1 | 39.40 |
|  | 2 | 40.19 |
|  | 3 | 41.55 |
| A50 | 1 | 39.90 |
|  | 2 | 38.93 |
|  | 3 | 40.75 |
| A100 | 1 | 35.65 |
|  | 2 | 35.96 |
|  | 3 | 36.09 |
| B0 | 1 | 28.91 |
|  | 2 | 28.93 |
|  | 3 | 28.66 |
| B10 | 1 | 32.22 |
|  | 2 | 32.06 |
|  | 3 | 32.14 |
| B50 | 1 | 31.72 |
|  | 2 | 31.84 |
|  | 3 | 31.99 |
| B100 | 1 | 34.26 |
|  | 2 | 34.44 |
|  | 3 | 34.20 |
| C | 1 | 28.41 |
|  | 2 | 28.16 |
|  | 3 | 28.53 |
| D | 1 | 29.36 |
|  | 2 | 29.31 |
|  | 3 | 29.33 |
| PCR-PC | 1 | 30.14 |
|  | 2 | 30.02 |
|  | 3 | 29.87 |
| Ex-NC | 1 |  |
|  | 2 |  |
|  | 3 |  |
| PCR-NC | 1 |  |
|  | 2 |  |
|  | 3 |  |

Table S6. amplification detail of Port Island Nov. 2018

| Sample name | PCR replicates | Cq |
| --- | --- | --- |
| N0 | 1 | 34.50 |
|  | 2 | 34.72 |
|  | 3 | 34.60 |
| N50 | 1 | 35.90 |
|  | 2 | 35.92 |
|  | 3 | 36.08 |
| N100 | 1 | 44.89 |
|  | 2 | 40.55 |
|  | 3 | 41.34 |
| N150 | 1 | 29.89 |
|  | 2 | 29.79 |
|  | 3 | 29.82 |
| N200 | 1 | 30.62 |
|  | 2 | 30.70 |
|  | 3 | 30.65 |
| N300 | 1 | 28.35 |
|  | 2 | 28.40 |
|  | 3 | 28.32 |
| S50 | 1 | 30.21 |
|  | 2 | 30.26 |
|  | 3 | 30.84 |
| S100 | 1 | 39.84 |
|  | 2 | 41.52 |
|  | 3 | 39.36 |
| S150 | 1 | 40.65 |
|  | 2 | 39.80 |
|  | 3 | 38.77 |
| S200 | 1 |  |
|  | 2 |  |
|  | 3 |  |
| S300 | 1 | 40.99 |
|  | 2 | 40.99 |
|  | 3 | 41.37 |
| W50 | 1 | 39.68 |
|  | 2 | 39.30 |
|  | 3 | 39.69 |
| W100 | 1 |  |
|  | 2 |  |
|  | 3 |  |
| W150 | 1 | 38.09 |
|  | 2 | 38.66 |
|  | 3 | 38.18 |
| W200 | 1 |  |
|  | 2 |  |
|  | 3 |  |
| W300 | 1 | 34.57 |
|  | 2 | 34.35 |
|  | 3 | 34.30 |
| E50 | 1 | 34.82 |
|  | 2 | 35.15 |
|  | 3 | 34.42 |
| E100 | 1 | 39.53 |
|  | 2 | 41.72 |
|  | 3 | 42.92 |
| E150 | 1 |  |
|  | 2 |  |
|  | 3 |  |
| E200 | 1 | 36.28 |
|  | 2 | 36.52 |
|  | 3 | 36.56 |
| E300 | 1 | 39.78 |
|  | 2 | 46.79 |
|  | 3 |  |
| PCR-PC | 1 | 29.52 |
|  | 2 | 29.78 |
|  | 3 | 29.86 |
| Ex-NC 1 | 1 | 41.25 |
|  | 2 |  |
|  | 3 |  |
| Ex-NC 2 | 1 |  |
|  | 2 |  |
|  | 3 |  |
| Ex-NC 3 | 1 |  |
|  | 2 |  |
|  | 3 |  |
| Ex-NC 4 | 1 |  |
|  | 2 |  |
|  | 3 |  |
| PCR-NC | 1 |  |
|  | 2 |  |
|  | 3 |  |

Samples of S200, E150, W100, and W200 could not be extracted successfully (see text for details).

Table S7. Accession numbers of target and coexistent species DNA data used for designing primers and probe

|  | Species | Accession number |
| --- | --- | --- |
| Target species | *Linepithema humile* | FJ16127, FJ161216, AF1147050, HQ207391, FJ4666879, FJ466680-4 |
| Non-target species | *Camponotus vitiosus* | AB019416 |
|  | *Formica japonica* | HQ619707, JQ350712, KX665091-4 |
|  | *Lasius japonicus* | AB371014-7 |
|  | *Ochetellus glaber* | JQ913605, JQ913622, DQ353333, EU127555 |
|  | *Pachycondyla chinensis* | KC685046-7, GQ264572, GQ264570 |
|  | *Pheidole noda* | AY762650, EF518375 |
|  | *Pristomyrmex punctatus* | EU342353-6 |
|  | *Temnothorax spinosior* | MF436658 |
|  | *Tetramorium tsushimae* | AY641691-700 |

Table S8. Result of preliminary comparison experiment between real-time PCR and LAMP assays

| Method | DNA template | Detection | Cq | SE |
| --- | --- | --- | --- | --- |
| Real-time PCR | ×10 | + | 26.20 | 0.08 |
|  | ×10^2 | + | 29.58 | 0.05 |
|  | ×10^3 | + | 32.79 | 0.05 |
|  | ×10^4 | + | 36.78 | 0.19 |
|  | ×10^5 | + | 41.25 | 0.39 |
|  | ×10^6 | – |  |  |
|  | ×10^7 | – |  |  |
| LAMP assay | ×10 | + |  |  |
|  | ×10^2 | + |  |  |
|  | ×10^3 | – |  |  |

**Methods for Table S8**

We compared detection sensitivity between two detection assays, real-time PCR and LAMP method. DNA was extracted from the tissue of the Argentine ant by alkaline extraction. First, 100 µl of NaOH (50mM) was added to the whole body of the Argentine ant. After incubating at 95 °C for 15 min, 100 µl Tris-HCl (0.2 M, pH 8.0) was added and centrifuged at 13,000 rpm for 5 min. The supernatant was then used as a DNA stock solution. In both assays, we used DNA templates made of the stock solution, diluted 10- to 10^7^- fold for real-time PCR, and 10- to 10^3^-fold for the LAMP assay.

For real-time PCR, we used the primers and a probe designed in this study, and performed in the same way as mentioned in the main text. For the LAMP assay, we used four primers reported by Ide et al. (2018). DNA amplification was performed using a Loopamp DNA Amplification Kit (Eiken Chemical, Tokyo, Japan). The reaction reagent mixture was 25 µl and the composition was as follows: 1×reaction Mix, 40 pmol of two primers, FIP and BIP, 5 pmol of the other two primers, F3 and b3, 1 µl Bst DNA Polymerase, 2 µl DNA sample, and ultrapure water. We detected LAMP products using LoopampEXIA® (Eiken Chemical). Since Cq values have little meaning in the LAMP assay, the results are shown only as positive or negative.

**Results for Table S8**

For real-time PCR, DNA was detected in 10- to 10^5^- fold dilutions, but not thereafter. DNA was negative in any negative control. For the LAMP assay, DNA was detected only in 10- and 10^2^- fold dilutions (Table S8).

Table S9. Number of times traditional surveys were conducted

|  | FM-1 | FM-2 | FM-3 | FM-4 |
| --- | --- | --- | --- | --- |
| PP traps | 12 | 12 | 2 | 18 |
| Sugar traps | 24 | 71 | 12 | 12 |
| Observations and hand samplings | 0 | 0 | 9 | 0 |
| Detection ratio of domestic ants | 22% | 4% | 89% | 40% |

Detection ratio indicates the number of surveys in which domestic ants were detected.

**Reference**

Ide, T., Kanzaki, N., Masuya, H., & Okabe, K. Application of the LAMP assay for the detection of the Argentine ant, *Linepithema humile* (Hymenoptera: Formicidae), from captures of pan traps. *Appl. Entomol. Zool.* **53**, 275-279 (2018).
